# Coronal and apical sealing ability of a new endodontic cement

**Published:** 2009-01-07

**Authors:** Morvarid Zafar, Maryam Iravani, Mohammad Jafar Eghbal, Saeed Asgary

**Affiliations:** 1 *Research Assistant, Dental Research Center, Shahid Beheshti University of Medical Sciences, Tehran, Iran.*; 2 *Department of Endodontics, Iranian Center for Endodontic Research, Dental Research Center, Dental School, Shahid Beheshti University of Medical Sciences, Tehran, Iran.*

**Keywords:** Dental leakage, Endodontics, Filling, Mineral trioxide aggregate, New material

## Abstract

**INTRODUCTION:** This *in vitro* study aims to evaluate the coronal and apical sealing ability of gutta-percha (GP) root filling used with either mineral trioxide aggregate (MTA), new endodontic cement (NEC) or AH26 as filler/sealers.

**MATERIALS AND METHODS:** Forty eight single-rooted extracted teeth were selected, decoronated and then instrumented. Samples were randomly divided into three experimental (n=12) and two control groups (n=6). In group 1, root canals were filled using lateral condensation technique (L); while single cone technique (S) was used for groups 2 and 3. AH26, MTA and NEC were the root canal sealer/fillers in groups 1, 2 and 3, respectively. Samples were immersed in 1% methylene-blue dye and then independently centrifuged apically and coronally. The roots were split longitudinally and linear extent of dye penetration was measured with a stereomicroscope from apical and coronal directions. Data were analyzed using One-way ANOVA and T-test.

**RESULTS:** No statistical differences in mean apical dye penetration between groups LGP/AH26, SGP/MTA and SGP/NEC were found; SGP/NEC group showed significantly less coronal dye penetration (P<0.001).

**CONCLUSION:** Considering the limitations of this *in vitro* study, it was concluded that the simple single cone technique with NEC can provide favorable coronal and apical seal.

## INTRODUCTION

Bacteria and their by-products are the greatest contributing factor to pulpal and periapical disease (1-2). Therefore all pathways between root canal system and periodontium should be filled or sealed with endodontic materials to prevent bacterial leakage. While several studies have emphasized the importance of coronal seal, a hermetic seal of both coronal and apical segments of root canal system are necessary to prevent bacterial leakage to ensure long-term clinical success (3-7). Coronal seal can deteriorate because of delayed permanent restoration, recurrent caries and fracture of the restoration or tooth crown (8-9). In such cases the exposed root canal filling materials remain the only barrier between oral environment and periradicular tissues.

Though many different filling materials and techniques have been proposed for root canal fillings, lateral condensation technique (L) employing gutta-percha (GP) has been the method of choice for over a century (10-11). Recent studies have suggested mineral trioxide aggregate (MTA) as a good alternative for root canal filling (10). MTA has been shown to be very effective in sealing the paths between root canal system and its surroundings (12). There are some studies indicating that MTA causes less leakage than amalgam, super-EBA and other materials when used as a root-end filling (13-15) or as intracoronal barriers to prevent coronal microleakage (16). Orthograde use of MTA for the entire root canal system has been suggested (17-19), but many researchers have cast doubts on the hermetic seal of this root canal filling material (20).

Recently, new endodontic cement (NEC; witch the other name is CEM cement) with different chemical composition (21-22) but identical applications as MTA has been developed (23-24) and proposed for root canal fillings. Although the *in vitro* studies (23,25) and *in vivo* vital pulp therapies (24,26) revealed comparable results for the sealing abilities of NEC and MTA, NEC offers some benefits over MTA such as improved handling, shorter setting time, lesser film thickness, better flow (21), an enhanced antibacterial effect (27) as well as a better ability to form hydroxyapatite in normal saline (28). Moreover, NEC promises to have an estimated lower cost.

The sealing ability of endodontic materials has been assessed by various methods such as dye or bacterial penetration, electrical methods, fluid filtration technique, radioisotope tracing and marginal adaptation by SEM; *in vitro* dye penetration studies have been carried out for decades as a simple effective method to evaluate the leakage (29).

The purpose of this study was to compare the coronal and apical dye penetration of a commonly used root canal treatment techniques ie laterally condensed gutta-percha (LGP) and AH26 sealer, with two other more novel techniques; single cone gutta-percha (SGP) used in conjunction with MTA, and SGP used with NEC as the simpler method.

## MATERIALS AND METHODS

Forty eight extracted single-rooted human teeth were used for this experimental study. All procedures were carried out according to protocols approved by the Dental Research Center, Shahid Beheshti University MC, during 2007. The teeth were examined and radio- graphed and samples with canal calcifications or anatomical abnormalities were excluded. They were stored in sterile normal saline solution and kept moist before and during the entire experiment. Decoronation was then performed at CEJ level using high speed fissure bur and water spray. The roots’ length ranged between 12-14 mm. Apical foramina size was not more than K-file size #30.

After etching coronal surface and apical portion of each root with 37% phosphoric acid, the two coronal and apical openings were covered with a “no mix” bonding resin (GAC International Inc., Bohemia, NY, USA) in order to seal the dentinal tubules and accessory canals. The root surfaces were covered with one layer of nail varnish within 2 mm of the apical foramen before canal preparation.

Patency of the apical foramen was confirmed by passing a K-Flex file size #10 (Mani, Japan) 1 mm through it before starting preparation. A K-Flex file size #15 was introduced into the canal of each sample till it was visible at the apical foramen. The working length was established by subtracting 1 mm from this length and reconfirmed by radiography. The apical portion of the root canals was prepared up to K-Flex file size #40 and then flared up to #80 using a step back technique. A K-Flex patency file size #15 was used for canal preparation phase. Normal saline solution was used as irrigation. Samples were randomly placed into five groups, three experimental groups (n=12) and two negative and positive control groups (n=6).

In group 1 (LGP/AH26), master gutta-percha cone size #40 (Ariadent, Tehran, Iran) was used for obturation. The canal walls were coated with AH_26_ sealer (*Dentsply,* DeTrey, Konstanz, Germany). The master cone was coated with sealer and reseated. Obturation was completed using lateral condensation technique.

In group 2 (SGP/MTA), ProRoot MTA (*Dentsply*, Tulsa Dental, Tulsa, OK, USA) powder and liquid was mixed according to the manufacturers’ instructions. This mixture was applied to the canal walls using a Lentulo spiral drill until the material reached the coronal orifice. Gutta-percha cone size #40 was then placed into the canal to within 1-2 mm of the working length. Excess gutta-percha and MTA cement was removed from the coronal portion of the root canal.

In group 3 (SGP/NEC), the group 2 procedure was repeated using NEC instead of MTA. The negative control roots were restored as group 1; the canals in the positive controls however, were prepared and left unfilled.

All groups were stored in 100% humidity at 37^º^ C for 7 days and they were again covered with a new layer of nail varnish so that only the apical foramen and canal’s orifice remained exposed. The negative controls however were completely covered.

**Figure 1:**
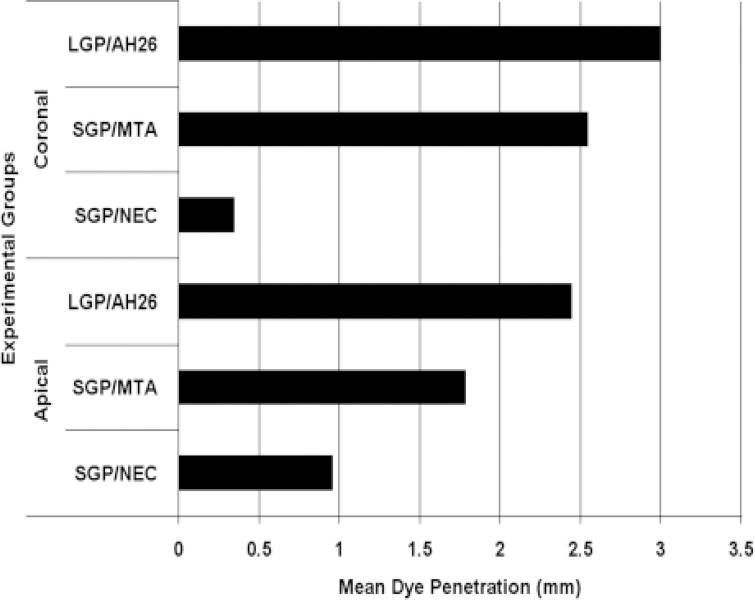
Bar chart showing the mean dye penetration values in three experimental groups in coronal and apical portions (L: lateral condensation technique, S: single cone technique, GP: gutta-percha, MTA: mineral trioxide aggregate, NEC: new endodontic cement).

All specimens were immersed in 1% methylene blue dye. Each tooth was centrifuged twice, once in coronal direction and then apically (3000 rpm in 5 min) to stimulate rapid dye penetration.

The roots were rinsed one hour in tap water and dried at room temperature for 24 hours. They were then split longitudinally with high speed handpiece and samples were viewed under a stereomicroscope (SZX9/12, Olympus, Tokyo, Japan) at ×12 magnification. Apical and coronal dye penetration was measured from the anatomical apex by an independent examiner to detect the maximum dye penetration in apical and coronal directions.

The data was collected and then subjected to a statistical analysis using One-way ANOVA and T-test with P<0.05.

## RESULTS

Experiment groups demonstrated dye penetration; the negative controls did not display this whereas all six positive controls showed total dye penetration through the entire length of the root canal.

The lowest coronal and apical dye penetration mean was observed in SGP/NEC group (0.342±0.405 and 0.958±1.656 mm), followed by SGP/MTA group (2.542±2.571 and 1.783±2.999 mm) and the LGP/AH26 group (3±3.542 and 2.442±2.875 mm). The mean leakage values in the experimental groups are represented in Figure 1.

The ANOVA test demonstrated statistically significant differences in dye penetration of experiment groups in coronal portions (P<0.001) and no difference in the apical segments. Pair comparison using T-test showed statistical difference in depth of coronal dye penetration between SGP/NEC and two other groups (P<0.001).

## DISCUSSION

There is a growing body of evidence that emphasizes the significance of apical and coronal seal in the success of endodontic treatment (30). Various studies have employed different methods to evaluate apical and coronal microleakage and there are as yet no concrete results that prove the superiority of one over the other. Arguably dye penetration method is a very popular technique for microleakage studies (31); the advantages include low cost, low toxicity, good availability and ease of storage (29). Torabinejad has reasoned that if a root filling material does not allow penetration of small particles such as dye molecules, it is more likely to have the potential to prevent microleakage of bacteria and their by-products (13). In this study, linear measurement of methylene blue dye penetration was used as the judgment criteria for coronal and apical seal.

MTA has proven to be an endodontic material of choice with potential for several clinical applications due to its superior sealing properties (13-15), for example its ability to set in the presence of moisture or blood (32) and biocompatibility (18,24,26). This is why some have suggested it as an orthograde obturating material for the entire root canal system (19-20). In this study, there was no significant difference in coronal and apical dye leakage between SGP/MTA and LGP/AH26. Al-Hezaimi *et al.* reported better sealing ability of MTA as a root canal filling material compared with gutta-percha (10), in contrast to Vizgirda *et al. *report*.* (20). The poor handling characteristics of MTA (21) and inappropriate insertion of the material into the entire root length (12-14mm) and diameter of the tooth (3-D) may be why MTA is an unpredictable root canal filling. On the other hand, in the single cone technique eliminating accessory gutta-percha allowed an improved seal as greater volumes of MTA or NEC was utilized. It is noteworthy to mention that the greater the volume, the greater the expansion of MTA and NEC during setting (21).

Our hypothesis stated that the good sealing/filling property of NEC specifically the coronal aspect is due to its physical and chemical properties (21-22). As predicted SGP/NEC group exhibited the lowest mean apical and coronal dye leakage values, however this difference was statistically significant in the coronal portion only. Although NEC showed positive dimensional change like MTA, its superior film thickness, flow, and setting time adds further advantages to NEC (21). Furthermore, NEC is able to produce hydroxyapatite with endogenous as well as exogenous ions (28). Once the communication pathway between root canal system and external area is blocked by the effective adhesion of the material and to the surrounding dentine, an additional seal is created for the external surface of the NEC and surrounding dentin (28).

In this study, we employed single cone technique in conjunction with MTA or NEC for root canal fillings. Usage of MTA for orthograde root canal filling has been previously suggested for limited number of cases such as one-step apexification. It has been recognized that for retreatment purposes, the canal should be filled with a material which can be re-entered easily following canal obturation for salvage failed endodontic treatment (33). Therefore, we recommended that orthograde root canal filling with MTA or NEC be performed with single cone technique so that clinicians can access the entire root canal in retreatment cases or periradicular surgery via the single cone gutta-percha technique.

## CONCLUSION

Although a complete hermetic apical seal cannot be achieved with either root canal filling materials or techniques; under the conditions of this *in vitro* study we concluded that easy single cone method using NEC provided acceptable coronal and apical seal.

Of significant clinical interest is the lowest apical and coronal leakage values achieved when using new endodontic cement with single gutta-percha master cone as a simple and easy technique. Although results obtained by *in vitro* sealing studies cannot be directly evaluated clinically, we can state that NEC is a promising root canal filling material with good sealing ability.
